# Editorial: Women in coaching and leadership

**DOI:** 10.3389/fspor.2025.1573237

**Published:** 2025-02-27

**Authors:** Áine MacNamara, Dave Collins

**Affiliations:** ^1^ Faculty of Science & Health, School of Health & Human Performance, Dublin City University, Dublin, Ireland; ^2^ Institute of Sport, Physical Education and Health Sciences, University of Edinburgh, Edinburgh, United Kingdom; ^3^ Grey Matters Performance Ltd., Stratford-upon-Avon, United Kingdom

**Keywords:** coaching, gender, female, management, performance

**Editorial on the Reasearch Topic**
Women in coaching and leadership

The landscape of sports, at all levels of performance, has witnessed a significant surge in female participation. Unfortunately, however, this increase has not been paralleled by a rise in women occupying coaching and leadership roles and the representation of female coaches remains low, especially in high-performance contexts. To this end, much attention has been paid to addressing the disparity between male and female coaches, with the development of strategies to support women in coaching and leadership positions evident across sporting contexts. In high-performance sport coaching, however, efforts to increase the number of female coaches must be complemented by an understanding of the specific skills, abilities, and support structures that *anyone*, male or female, needs to achieve in these positions. In short, appointments must be performance based to ensure fair levels of support for highly committed athletes.

As such, we would argue that simply striving for equal numerical representation of males and females is not the correct priority focus. Instead, consideration of the systemic barriers that women face in elite coaching environments, such as limited access to mentorship, unequal professional development opportunities, and the biases that challenge their credibility may more appropriately impact practice. Moving beyond numerical targets toward development strategies will ensure that women in coaching are not only present but also acknowledged and empowered to contribute and develop meaningfully.

With this in mind, this Research Topic exploring Women in Coaching and Leadership represents a move away from a focus on gender-based numeric disparity. In contrast, it represents a move towards a consideration of the factors, and potential solutions, to supporting women in coaching and leadership. For example, *Firsts in their field: the perceptions of women who have led the way* (Stone et al.) explores the experiences of six women who were pioneers in holding high-level positions within sports organisations. The study highlights that, while gender-based challenges were historically present, the combination of leadership abilities, effective relationship-building, and personal resilience can enable women to break new ground in sports leadership. The experiences of these trailblazing women offers valuable insights for future generations aiming to navigate and succeed in similar environments.

In similar fashion, the article titled *Using the coaches voice to improve the representation and experience of females in coaching: a Gaelic games perspective* (Haughey et al.) examines the underrepresentation of female coaches in Gaelic games but moves beyond purely descriptive accounts to a consideration of the strategies that enhanced their participation and experiences. Recognising the predominantly male-dominated context of coaching in Gaelic Games, particularly in leadership roles, the female coaches in this study noted that traditional gender roles, organisational structure, and societal expectations hindered their coaching aspirations. The female coaches in this study noted that targeted strategies such as mentorship, inclusive policies, and tailored training created a supportive environment that encouraged their participation in coaching.

Reflecting the importance of targeted programmes to address the underrepresentation of female coaches, *Women Coaches Leadership Development Programme: An Evaluation Study of Programme Effectiveness* (Jowett et al.) examined the impact of a leadership development program. Interestingly, and reflecting this Research Topic, this study challenges the assumption that there are inherently “female solutions” to “female problems” and how this approach can oversimplify the complexity of gendered experiences in domains such as leadership and sport coaching. Women are not a homogenous group; their challenges, opportunities, and responses are shaped by intersecting factors such as context, sport, culture, and personal background. As such, although the findings of this study demonstrated the effectiveness of a leadership development programme in enhancing leadership skills, confidence, and career trajectories among female coaches, solutions to inequities cannot be universally applied to all women, as their experiences vary widely based on context and environment. For example, while mentorship programs may benefit some, it is unlikely to be the only factor that supports retention and progression of female coaches. As such, any strategy must address the nuanced realities that different women face in different contexts. The most effective strategy requires individualized, context-sensitive interventions that consider the diverse and dynamic nature of women's experiences in coaching and leadership.

Of course, the progression of women in high-performance sport contexts is inherently complex and cannot be resolved solely by focusing on individual development. While the studies presented in this Research Topic present useful and interesting insights into the experiences of females in coaching and leadership, and the effectiveness of development programmes in supporting female coaches and leadership, initiatives such as mentorship programs, leadership training, and confidence-building workshops. While valuable, however, these fail to address the structural and systemic barriers that act to influence women's retention and progression in coaching and leadership. Deeply embedded organisational structures, including hiring practices, funding disparities, and work-life balance expectations, place disproportionate burdens on women in sport coaching and leadership positions. It can also be argued that it is not possible to address and solve these challenges without institutional change, including policy reforms, changes to resource allocation, and shifts in attitudes towards women in leadership roles. Without systemic transformation, the focus on individual agency risks placing responsibility on women to overcome obstacles that are, in reality, embedded within the wider high-performance sport ecosystem.

In conclusion, while significant strides have been made to support and elevate women in coaching and leadership roles, there remains a considerable journey ahead. Continued investment in leadership development programs, organizational initiatives, and cultural transformation is essential to achieve true gender parity in sports coaching and leadership. By fostering an environment that values and supports female coaches, the sports industry can harness a broader range of talents and perspectives, ultimately enriching the sporting experience for all.

We are grateful for the excellent contributions made by these authors.

